# Non-linear effects of non-host diversity on the removal of free-living infective stages of parasites

**DOI:** 10.1007/s00442-023-05462-2

**Published:** 2024-02-01

**Authors:** Jennifer E. Welsh, Mirjana Markovic, Jaap van der Meer, David W. Thieltges

**Affiliations:** 1 Department of Coastal Systems, NIOZ Royal Netherlands Institute for Sea Research, PO Box 59, 1790 AB Den Burg, The Netherlands; 2 Wageningen Marine Research, Korringaweg 7, 4401 NT Yerseke, The Netherlands; 3 Aquaculture and Fisheries Group, Wageningen University and Research, Wageningen, The Netherlands; 4 Groningen Institute for Evolutionary Life-Sciences, GELIFES, University of Groningen, Nijenborgh 7, 9747 AG Groningen, The Netherlands

**Keywords:** Disease ecology, Dilution effect, Biodiversity, Parasite removal, Transmission

## Abstract

Among the ecological functions and services of biodiversity is the potential buffering of diseases through dilution effects where increased biodiversity results in a reduction in disease risk for humans and wildlife hosts. Whether such effects are a universal phenomenon is still under intense debate and diversity effects are little studied in cases when non-host organisms remove free-living parasite stages during their transmission from one host to the next by consumption or physical obstruction. Here, we investigated non-host diversity effects on the removal of cercarial stages of trematodes, ubiquitous parasites in aquatic ecosystems. In laboratory experiments using response surface designs, varying both diversity and density at same time, we compared three combinations of two non-hosts at four density levels: predatory crabs that actively remove cercariae from the water column via their mouth parts and gills, filter feeding oysters that passively filter cercariae from the water column while not becoming infected themselves, and seaweed which physically obstructs cercariae. The addition of a second non-host did not generally result in increased parasite removal but neutralised, amplified or reduced the parasite removal exerted by the first non-host, depending on the density and non-host combination. These non-linear non-host diversity effects were probably driven by intra- and interspecific interactions and suggest the need to integrate non-host diversity effects in understanding the links between community diversity and infection risk.

**Supplementary Information:**

The online version contains supplementary material available at 10.1007/s00442-023-05462-2.

## Introduction

The ongoing biodiversity crisis has sparked intensive research on the role of species diversity for the functioning of ecosystems and for the goods and services they provide to humans (Hooper et al. 2005; Cardinale et al. 2012). A broad range of correlative and experimental studies has revealed that biological diversity is a pivotal driver of ecosystem functions such as nutrient cycling, carbon storage, and productivity (Cardinale et al. 2006; Tilman et al. 2014; Duffy et al. 2017). Many of these ecosystem functions are also directly or indirectly beneficial for humans, either for production of resources such as food (provisioning services) or the regulation of environmental processes such as soil genesis, pollination and climate regulation (regulating services; Cardinale et al. 2012). Among the latter, disease control has recently emerged as a potential additional ecosystem service of biodiversity. This idea relates to a phenomenon called the *dilution effect*, which is the theoretical concept that increased biodiversity results in a reduction in disease risk for humans and also more broadly for wildlife hosts (Keesing et al 2006; Ostfeld and Keesing 2012).

While there are several mechanisms that can lead to a biodiversity-mediated alteration of disease risk (Keesing et al. 2006), the mechanism receiving most attention has been *encounter reduction,* which is when surrounding communities cause a reduction in encounters between susceptible and infectious hosts or with infective stages. The most prominent examples of encounter reduction come from vector-borne diseases with frequency-dependent transmission, such as Lyme disease, in which hosts of lower competence can act as decoys for vectors and pathogens. Thereby, the pathogen pool becomes diluted, leading to reduced prevalence in focal hosts and consequently lowering human exposure (Ostfeld and Keesing 2000, 2012). A second line of research expanded on the encounter reduction theory by assessing how changes in the diversity of hosts with different competence affect non-vector borne diseases with density-dependent transmission. Most of this work has been conducted with free-living cercarial stages of trematodes that infect tadpoles and it has indicated that less competent hosts can act as decoys for infective stages, thereby lowering infection levels in the main competent host (Johnson et al. 2008a, 2013). Finally, a third line of research has been focussing on how non-hosts (i.e., organisms which do not serve as competent host or less competent decoys and thus do not become infected) can interfere with the transmission of free-living infective stages (Thieltges et al. 2008; Johnson and Thieltges 2010; Koprivnikar et al. 2023). This interference can, for example, occur when non-hosts prey on free-living infective stages or act as a physical obstruction. The resulting removal of parasites from the pool of infective stages subsequently leads to reduced infection levels in focal hosts (Johnson and Thieltges 2010; Johnson et al. 2010; Orlofske et al. 2012; Goedknegt et al. 2015). This form of transmission interference is probably widespread and does not only affect free-living infective stages of macroparasites but also inhibits the transmission of microparasites such as viruses (Welsh et al. 2020).

Although there is no doubt that biodiversity can affect disease transmission and dynamics via the mechanisms discussed above, whether a reduction in disease risk with an increase in diversity (dilution effect) is a universal phenomenon or whether diversity effects are instead idiosyncratic is still under intense debate (Randolph and Dobson 2012; Lafferty and Wood 2013; Ostfeld and Keesing 2013; Salkeld et al. 2013; Wood and Lafferty 2013; Civitello et al. 2015; Johnson et al. 2015; Halsey 2019; Halliday et al. 2020; Rohr et al. 2020; Keesing and Ostfeld 2021). In the case of vector-borne diseases such as Lyme disease, dilution effects have been reported from several disease systems, including aquatic and terrestrial systems (Ostfeld and Keesing 2012). However, the mechanisms linking biodiversity and disease risk for focal hosts may actually be more complex and may result not only in a reduction but also in an amplification of disease risk, depending on the specific circumstances such as habitat changes, host densities and spatial scales (Wood and Lafferty 2013; Cohen et al. 2016; Halliday and Rohr 2019; Rohr et al. 2020). Therefore, experimental manipulations rather than field observations have been better suited to disentangle the effects of host competency on disease risk, and have especially been applied in the case of parasites with density-dependent transmission (Johnson et al. 2013, 2015). In addition, experiments also allow for the separation of true diversity effects from density effects as simply increasing the total density of host species may have the same effect as increasing the diversity of host species with differential competence. For example, by controlling for potential density effects through keeping total host density constant, experiments with the trematode *Ribeiroia ondatrae* infecting amphibians revealed a true diversity effect in form of a decline in tadpole infection levels in the presence of another amphibian species with lower host competence (Johnson et al. 2008a).

While experimental studies on the joint effects of the diversity and density of less competent decoy hosts on disease risk have been increasing, experimental studies on diversity effects regarding the removal of infective stages by co-occurring non-host species are still limited. In general, studies have shown that a multitude of non-host species can consume or obstruct infective stages of a large range of parasite groups from different environments (Thieltges et al. 2008; Johnson and Thieltges 2010; Johnson et al. 2010). Especially in aquatic environments, where vectors are much less common than on land and where transmission of parasites and pathogens mainly occurs via the water column, ‘pathogen filtration’ by non-hosts in form of physical obstructions by vegetation or filter feeding removal by bivalve beds has received increased interest as a potential ecosystem service and disease managing approach (Burge et al. 2016; Lamb et al. 2017; Klohmann and Padilla-Gamiño 2022). However, non-host species differ in their capability to consume or obstruct infective stages depending on the specific parasites or pathogens in question and some non-host species may not show an effect at all (e.g., Hopper et al. 2008; Orlofske et al. 2015; Welsh et al. 2014, 2020; Koprivnikar et al. 2023). Non-hosts that do consume or physically obstruct infective stages usually do so in a density-dependent fashion, i.e., the effect increases with non-host density (Thieltges et al. 2009; Rohr et al. 2015). However, whether the addition of other non-hosts results in true diversity effects or only simple density effects has been poorly studied to date. Diversity effects could result from differential effects of non-hosts on infective stages leading to additive effects, e.g., when one non-host species obstructs infective stages lower in the water column while another consumes stages from higher in the water column, resulting in elevated removal in two- versus single-species settings. Alternatively, diversity effects could arise from species interactions between non-hosts, such as interference competition or predation, e.g., when one non-host species affects the magnitude of infectious stages consumption or obstruction by another non-host, leading to lower removal in multi- compared to single-species settings. Partly, the lack of such diversity studies is probably related to the methodological difficulties in conducting meaningful comparisons of non-hosts of very different morphologies, sizes and parasite consumption or obstruction mechanisms (Johnson and Thieltges 2010). In any case, this scarcity of studies hampers our broader understanding of the mechanisms underlying the general relationship between biodiversity and disease (Johnson et al. 2015; Rohr et al. 2020; Keesing and Ostfeld 2021).

**Fig. 1:**
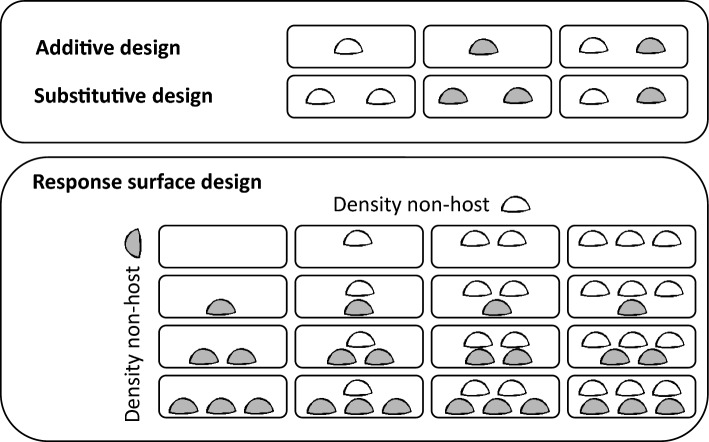
Differences between additive and substitutive experimental designs (above) and the response surface experimental design used in this study (below). The white and grey symbols indicate two different non-host species, the small rectangles individual experimental units. In our experiments, we used a two-factorial response surface design, with two non-host species and four density levels each

In this study, we used an experimental approach from general community ecology, the response surface design, to overcome methodological issues in disentangling diversity and density effects of communities of different non-host taxa organisms with different parasite consumption or obstruction mechanisms. Typically, response surface design experiments incorporate two different competitive species at various densities and thus combine additive and substitutive experimental designs (Inouye 2001; Fig. 1). This design allows for the statistical testing of inter- and intraspecific interactions and is, therefore, suitable to disentangle the effects of species diversity from density effects. For our laboratory experiments, we used cercariae of a common marine trematode species (*Himasthla elongata*), a parasite with a complex life cycle. This species uses periwinkles (*Littorina littorea*) as first intermediate hosts, from which cercariae (with positive geotaxis and phototaxis; Nikolaev et al. 2017; Correia et al. 2021) are released into the water column and subsequently infect (as metacercarial cysts) a second intermediate bivalve host, such as the blue mussel *Mytilus edulis.* After the predation of the bivalve host by a definitive bird host, the parasite develops into its adult stage and sexually reproduces inside the bird, after which eggs are released with the host’s faeces (Werding 1969). Infection intensity in the second intermediate mussel host is generally dose-dependent, i.e., the number of metacercarial cysts in a mussel is positively correlated with the number of infective cercarial stages it has been exposed to (Liddell et al. 2017). Hence, any alterations of the number of infective cercarial stages by non-hosts in the environment surrounding mussels can, in principle, affect the risk of mussels becoming infected with metacercarial stages. In cases where this differential infection risk leads to altered transmission and metacercarial infection levels, this will have fitness consequences for mussels, e.g., reduced condition (Bommarito et al. 2022). Such negative effects of metacercarial infections in mussels are generally considered to be density-dependent (Thieltges 2006; Stier et al. 2015).

To study potential diversity effects of non-host diversity on parasite removal, we used three non-host species from widely different taxa that are common in coastal waters, in particular on mussel and oyster beds, and that have been shown to interfere with cercarial transmission via different mechanisms: the predatory shore crab *Carcinus maenas* actively removes cercariae from the water column via its mouth parts and gills (Welsh et al. 2019); the filter feeding Pacific oyster *Crassostrea gigas* passively filters cercariae from the water column while not becoming infected itself (Thieltges et al. 2008; Welsh et al. 2019); and the seaweed *Sargassum muticum* physically traps cercariae (Welsh et al. 2014). Crabs are a known predator of Pacific oysters and can affect filtration activity of bivalves through anti-predator responses of their potential prey and through physical interference (Mascaró and Seed 2000; Mascaro and Seed 2001; Cornelius et al. 2023). We hypothesised that the addition of a second non-host species will result in additive diversity effects in crab/seaweed and seaweed/oyster combinations through elevated parasite removal from the pool of infective stages due to different consumption and obstruction mechanisms. For crab/oyster combinations, we hypothesised interactive diversity effects through lowered removal rates in two-species settings due to interference interaction. Instead of using the infection levels in second intermediate hosts to identify non-host diversity effects related to parasite removal, we determined the number of remaining free-living parasite stages after removal of the non-host communities. Therefore, the results were not confounded by direct predation on second intermediate hosts by non-hosts or non-consumptive effects such as behavioural changes of parasites and second intermediate hosts in presence of non-hosts.

## Materials and methods

### Experimental organisms

To obtain sources of cercariae, we collected periwinkles (*Littorina littorea*) from the intertidal in the vicinity of the NIOZ Royal Netherlands Institute for Sea Research on Texel (Wadden Sea, The Netherlands). Snails infected with *Himasthla elongata* were identified by shedding trials (release of cercariae under light at 27 °C), kept in aerated flow-through aquaria and fed with sea lettuce (*Ulva lactuca*). For the experiments, we obtained cercariae from infected snails by incubating approximately 30 snails under light at 27 °C in 3 L of seawater for 3 h. The required amount of cercariae was then pipetted and administered to the experimental units within one hour (i.e., cercariae were not older than 4 h at the start of the experiment).

Three non-host species which all coexist in the study area, in particular on mixed mussel and oyster beds (see Electronic Supplementary Material ESM1 for more information on the habitat), were used: Pacific oysters (*Crassostrea gigas*) which are sessile filter feeders, shore crabs (*Carcinus maenas*) which are motile active predators and seaweed (*Sargassum muticum*), which forms a physical obstruction and traps cercariae. The sizes of non-hosts reflected common size ranges observed in the field: Pacific oysters: 10.5 ± 1.2 cm widest diameter*,* shore crabs: 3.1 ± 0.3 cm carapace width, and seaweed: floating branches of individual plants. All three species were collected from the intertidal and shallow subtidal in the vicinity of the NIOZ Royal Netherlands Institute for Sea Research on Texel (Wadden Sea, The Netherlands). Immediately after collection, any epibionts were carefully removed and all organisms were kept in aerated flow through aquaria in the same climate chamber at 18 °C. Crabs were fed on a diet of mussels while oysters were fed algal bivalve feed (*Isochrysis galbana*, Instant Algae by Reed Mariculture Inc. USA; 4.1 billion cells ml^−1^; administered as 4 drops of algal feed per oyster, as recommended by Reed Mariculture).

### Experimental design

To test for the effects of non-host diversity on the removal of cercariae we used a two-factorial response surface design, with two different non-host species and four density levels. This design combined both additive (varying non-host diversity but also density at the same time) and substitutive (varying diversity but keeping density constant) designs and allowed us to separate diversity from density effects as well as identify potential interactive effects between both factors (Inouye 2001; Fig. 1). Density levels of the three non-host species reflected densities that can be locally observed in the field (see Electronic Supplementary Material ESM 1 for details) and were as follows: oysters (0, 1, 2, 6 ind.), crabs (0, 1, 2, 3 ind.), and seaweed (0, 5, 15, 30 g fresh weight after gently drying with a paper towel). The treatment with zero densities of non-host species served as a control for potential background losses of cercariae.

Three experiments were carried out in three separate runs in a temperature- and light-controlled room (18.0 °C ± 0.2 °C; 10:14 h light/dark cycle). Each experiment tested two different non-host species (oyster-crab, oyster-seaweed, crab-seaweed) at four density levels and each treatment was replicated four times (i.e., 64 replicate units in total per experiment; Fig. 1). Each of the replicate units consisted of a 2 L aquarium with 1500 ml of filtered seawater. To allow for acclimation, all test organisms were starved and kept in the experimental containers for 24 h prior to the experiment starting. At the start of the experiment, 100 cercariae were added to each experimental unit and the aquaria were left undisturbed for the following 3 h. After 3 h, the experiment was terminated by quickly removing all non-hosts with forceps. The water from each experimental unit was filtered through a 25 μm sieve to retain any remaining cercariae. The units were then flushed with filtered seawater and sieved a further two times to reduce chances of cercarial adhesion to the walls of the units. Subsequently, the cercariae were washed from the sieve and fixed using 10 ml of 96% ethanol and stained using Rose Bengal. After a minimum of 24 h to allow sufficient staining, all cercariae were counted in Petri dishes under a stereo microscope.

### Data visualisation and statistical analysis

To visualise the results, we plotted the mean absolute numbers of cercariae remaining per treatment combination by placing the different density levels of one of the two non-host species on the *x*-axis and separating the different densities of the second non-host species into four different line series. Although combining data points with lines in a categorical design is usually not appropriate, this graphical depiction allowed for an easy visualisation of potential interactions between the two main factors. Parallel lines would suggest additive effects of a second non-host species while crossing or diverging lines would indicate interaction effects of the two non-host species on cercarial removal.

The effects of non-host species on cercarial removal were investigated using binomial Generalized Linear Models (GLMs) with a log-link. We assumed a *linear pure death process* (i.e., each cercarial removal by non-hosts is an independent event) so that the number of cercarial stages remaining at the end of the experiment is binomially distributed, with a probability of cercariae surviving until the end being equal to *p* = *e*^−*θt*^ where *θ* is the rate at which cercariae are removed per unit of experimental time, and hence *t* = 1. This cercarial removal rate was assumed to be a function of non-host diversity, density and the interaction between both, thus:

$$\theta_{i,j} = \mu + \alpha_i + \beta_j + \gamma_{i,j}$$

where *α*_*i*_ represents the effect of the first non-host at the *i*th density, *β*_*j*_ of the second non-host at the *j*th density, and *γ*_*i*,*j*_ their interaction.

We then fitted a series of GLMs from the most complex to the least complex model (for an illustration of the model selection procedure see Electronic Supplementary Material Fig. S2). In the most complex model, all explanatory variables were included (including the interaction) while the simplest model only contained the intercept (null model). Using analysis of deviance, we identified the best fitting model by testing for significant differences between models of decreasing complexity. To illustrate the procedure, the most complex model was tested against the next less complex model (including the effects of both species but not their interaction). The difference in deviance (delta deviance; Δ Dev) between the two models was then divided by the dispersion factor (*ϕ*; most complex model residual deviance divided by degrees of freedom) and compared to the delta degree of freedom *χ*^2^ at 0.05 to identify statistical significance. A significant difference between two models indicated a better fit of the more complex model. Using the model coefficients and unique estimates of intercepts for each of the factors included in the best fitting model, we calculated cercarial removal rates and parasite survival (%). All analyses were carried out using R (R Core Team 2019) version 3.0.2 in RStudio (RStudio Team 2018). Raw data of all experiments can be found in Welsh et al. (2023).

## Results

### General patterns

**Fig. 2:**
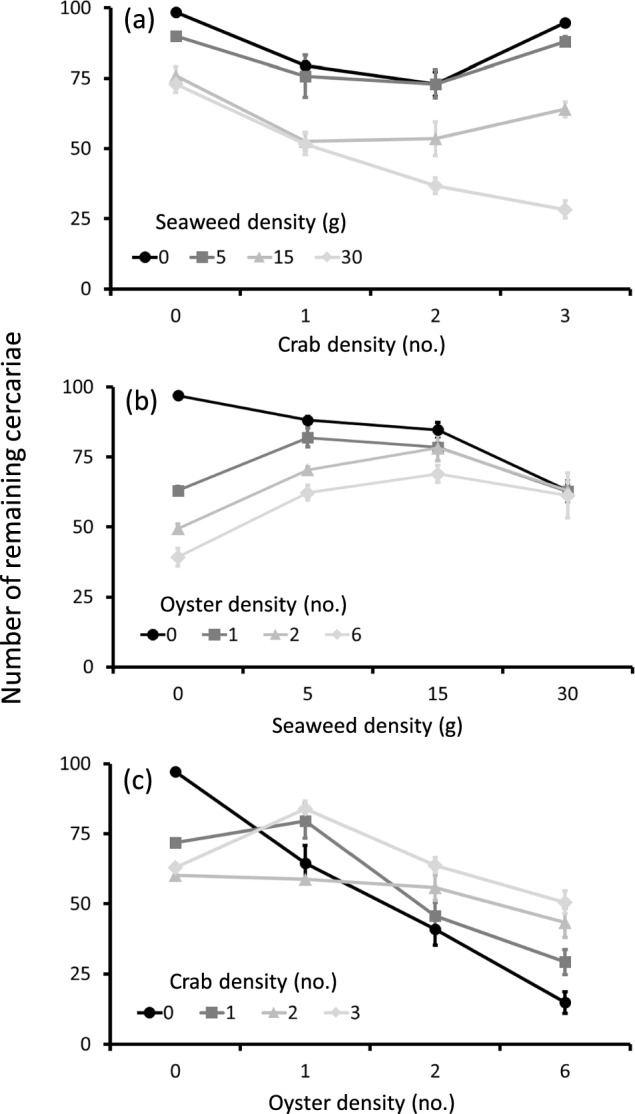
Mean number of infective cercarial stages remaining (± SE) after exposure to combinations of two non-host species at different densities in response surface design experiments with **a** crabs and seaweed, **b** seaweed and oysters, and **c** oysters and crabs. Each treatment combination was replicated four times, i.e., total *N* = 64 per experiment. Note that raw data are presented here to indicate the structure and variance of the data on which the analyses were based. Removal rates by non-hosts and cercarial survival were calculated from the best fitting models (Tables S1–3). For details, see main text

For all three combinations of non-host species, the best-fitting models were the most complex ones which included the interaction between both non-host species. Thus, the effect of a specific non-host species on cercarial removal depended on the density of the other non-host species (Table 1; Fig. 2, Electronic Supplementary Material Table S1). Data visualisation by plotting the mean absolute numbers of cercariae remaining at different non-host density levels for one species against that of the second species (Fig. 2) revealed diverging, converging and crossing of the lines, thus denoting the presence of interactions between the first and second non-host species. Therefore, depending on the non-host species combination and the density levels, the presence of a second non-host species resulted in a neutralisation, amplification or reduction of the parasite removal effects exerted by the first non-host species. In the treatments without non-hosts added, on average, 97–98.5% of the cercariae added were retrieved, indicating low cercarial losses during sieving and counting (Fig. 2; Electronic Supplementary Material Tables S2–4).

**Table 1:** Model selection results, showing the degrees of freedom (*df*) and deviances for each model from the most complex (model 1) to the simplest model (model 5) for each non-host species combination

Model code	Model	df	Deviance		
			Crab and seaweed	Seaweed and oyster	Oyster and crab
1	*X*_1_ + *X*_2_ + *X*_1_:*X*_2_	48	130.7	840.1	183.1
2	*X*_1_ + *X*_2_	57	306	1034.7	638.8
3	*X*_1_	60	615.3	1873.3	683.4
4	*X*_2_	60	1090.3	1232.3	1302
5	1	63	1373.9	1930.5	1355.5
ϕ from best fitting model			2.72	17.5	3.81

The best model explaining the number of cercarial stages remaining at the end of the experiment in all three cases was the most complex model which included densities of the first non-host species (*X*_1_), densities of the second non-host species (*X*_2_) and the interaction (*X*_1_:*X*_2_) between the two non-hosts species. The dispersion factor (*ϕ*) for the best-fitting model for each non-host species combination is shown. For details of model selection procedures see text and Fig. S1

### Crabs and seaweed experiment

In the experiment using crabs and seaweed, the mean number of infective stages remaining at the end of the experiment decreased with increasing crab and seaweed density (Fig. 2a). At low densities of crabs (0–1 ind.), the addition of seaweed to the experimental units had an additive effect (suggested by the roughly parallel lines), while at higher crab densities (2–3 ind.) survival of cercariae strongly decreased with increasing seaweed densities (diverging lines; Fig. 2a). The survival of cercariae was lowest (28%) and thus the total removal rate by the non-hosts highest (1.26; calculated from the best-fitting model; Electronic Supplementary Material Table S2) in the treatment with the highest crab and seaweed density levels (Fig. 2a).

In the absence of seaweed, increasing crab densities lead to a decrease in the number of cercariae remaining, however, at the highest crab density level (3 crabs), cercarial survival increased again, leading to a trough-shaped curve in the numbers of cercariae remaining (Fig. 2a; Electronic Supplementary Material Table S2). In the absence of crabs, cercarial survival decreased with increasing seaweed density, however, at the highest seaweed density level (30 g) cercarial survival was relatively similar to the one observed at the second highest density level (15 g; 73% and 76%, respectively; calculated from the best-fitting model; Electronic Supplementary Material Table S2).

### Seaweed and oysters experiment

In the experiment investigating the effects of seaweed and oysters, in absence of oysters the mean number of surviving infective stages remaining at the end of the experiment decreased with increasing seaweed density (Fig. 2b). Likewise, when seaweed was absent, the number of remaining cercariae decreased with increasing oyster densities (Fig. 2b).

However, when oysters and seaweed were combined, total cercarial removal rates were lower than in oyster-only treatments. Already adding the lowest seaweed density (5 g) to experimental units containing oysters lead to lower cercarial removal than in oyster-only settings at all three oyster densities (Fig. 2b). The difference in cercarial removal between seaweed-only and mixed treatments decreased with increasing seaweed densities (converging lines; Fig. 2b). At the highest seaweed density, cercarial survival was similar (~ 60%) across all oyster density treatments (calculated from the best-fitting model; Supplementary Electronic Material Table S3) as the seaweed-only treatment (Fig. 2b).

### Oysters and crabs experiment

Finally, in the experiment investigating oysters and crabs we observed a crossing of the different lines (Fig. 2c). In absence of crabs, the mean number of surviving infective stages remaining at the end of the experiment decreased with increasing oyster density, however, the addition of crabs to the experimental units resulted in a much lower effect of oyster density on cercarial removal (Fig. 2c). Likewise, while the mean number of surviving infective stages remaining at the end of the experiment decreased with increasing crab density in absence of oysters, this pattern was fully reversed at the highest oyster density (6 ind.), with cercarial removal decreasing with increased crab density and thus the highest cercarial survival was observed at the highest crab density (Fig. 2c; Electronic Supplementary Material Table S4).

As in the experiment with crabs and seaweed, cercarial survival in absence of the second non-host species decreased with crab density but then, albeit less strongly, increased again at the highest crab density (3 crabs; 63%, calculated from the best-fitting model; Electronic Supplementary Material Table S4) compared to the survival at the intermediate crab density (2 crabs; 60%; Electronic Supplementary Material Table S4; Fig. 2c).

## Discussion

In all three experiments, total parasite removal from the water column by the experimental non-host communities was a function of the interactive effects between the two non-host species. However, adding a second non-host species to the experimental units did not universally result in the increase (oyster-seaweed, crab-seaweed) or decrease (oyster-crab) of removal rates in two-species settings that we had hypothesized. Instead, increasing non-host diversity by adding a second species led to a neutralisation, amplification or reduction of the parasite removal effects exerted by the first non-host species, depending on the respective densities and non-host species combination. This suggests that diversity effects, in respect to parasite removal by non-hosts, exist independent of density effects but that the direction and magnitude of these effects are strongly conditional on the respective non-host combinations.

These non-linear effects of non-host diversity on parasite removal probably resulted from interactions among non-host species arising in the different non-host combinations. We had expected such interactions for the crab and oyster combination, however, in the other combinations adding a second non-host species to experimental units probably also affected the behaviour of one or both non-host species. In the case of the crab-seaweed combination, it is likely that the addition of seaweed to the experimental units allowed the crabs to move through the seaweed matrix. This way they could access cercarial stages which were swirling higher up in the water column that would otherwise have been unreachable if no seaweed were present. As higher densities of seaweed provided a denser matrix for the crabs, which themselves removed cercariae in a density-dependent fashion, removal rates in the combined treatments increased with increasing crab and seaweed densities and community removal rates were highest in the treatments with the highest crab and seaweed densities. Hence, through such interspecific interactions, the addition of seaweed may have strongly amplified the parasite removal effect exerted by crabs alone. In contrast, in the oyster-seaweed combination, the matrix created by the seaweed did not result in increased parasite removal by oysters, instead with an increase in seaweed density the seaweed floating in the experimental units probably trapped more and more cercariae so that fewer cercariae made it to the bottom of the experimental units where the oysters were positioned. Alternatively, branches of seaweed may occasionally have touched the bivalves’ mantle tissue, thereby disturbing their filtration. Hence, in this case, the addition of seaweed lead to a neutralisation of the parasite removal effects exerted by oysters alone. Finally, in the case of the oyster-crab combination, the addition of crabs lead to a reduction of the parasite removal effects exerted by oysters alone, possibly because the movements of crabs throughout the experimental units disturbed the oysters inducing valve closure and thus reduced their filtration activity. The interspecific interaction between oysters and crabs likely increased with crab density. This would explain our observation that, at the highest oyster density, cercarial survival was highest at the highest crab density and thus cancelled the pattern of highest cercarial survival at lowest crab densities when oysters were absent. We acknowledge that further experiments will be needed to verify the suggested interactions, however, it is plausible that differential behavioural changes initiated by the addition of a second non-host species underlie the non-linear diversity effects observed in our experiments.

In addition to interspecific interactions, intraspecific interactions among individuals of the same non-host species may have further modified total parasite removal rates in the experimental communities. For example, crabs showed slightly lower parasite removal rates at high densities compared to intermediate crab densities (albeit less pronounced in one of the two experiments with crabs). This may have resulted from intraspecific interactions among crabs which are known to show aggressive display and fighting behaviour in the presence of conspecifics, which can lead to reduced predation rates due to interference competition, often mediated by the habitat context which the seaweed may have provided (Griffen and Byers 2006; Smallegange et al. 2006). Similar interference interactions may have occurred in our experiments leading to a reduction in parasite removal rates at high crab densities. Such intraspecific interactions may also have an individual component as individual crabs differ in competitive strength (Sneddon et al. 2000) and this may explain the slight variation in removal rates at the highest crab densities between the two experiments involving crabs. Whatever the exact mechanisms, our experiments clearly indicated that non-host diversity effects resulted in different directions and magnitudes in the three non-host combinations.

The different non-host diversity effects observed in the experiments can be expected to result in a differential risk for the downstream mussel hosts to become infected through altering their exposure to infective stages. Given that infection levels in the second intermediate host are dose-dependent for the parasite-host system used in our experiments (Liddell et al. 2017), any changes in exposure are likely to translate into changes in transmission and infection levels, with fitness consequences for the mussel hosts due to the intensity-dependent negative effects of metacercarial infections (Thieltges 2006; Stier et al. 2015; Bommarito et al. 2022). Hence, depending on the outcome of the effects of intra- and interspecific interactions between non-host species on the total parasite removal, focal hosts are likely to experience very different parasite exposure and associated disease risks. This conclusion seems to contradict findings from meta-analyses based on published studies on diversity effects on disease risk which found evidence for the generality of dilution effects among diverse host and disease systems (Civitello et al. 2015; Huang et al. 2017). However, the database underlying these analyses included a broad variety of studies, many of which only studied the effect of the addition of a single less competent host species on parasite transmission rather than effects of more complex communities of less competent hosts or non-hosts. In addition, most of these studies were not designed to disentangle diversity from density effects. Studies that tried to separate diversity from density effects are surprisingly rare and paint a more complex picture. Some studies that investigated diversity effects of hosts of differential competence found diversity effects for both fungal pathogens and trematodes infecting amphibian hosts (Johnson et al. 2008b; Searle et al. 2011). In contrast, experiments using substitutive designs (i.e., varying diversity while keeping density constant) on cercarial predation by various non-host species did not reveal diversity effects (Rohr et al. 2015). However, in a mescosom experiment of the same study which included snails as sources for cercariae, the odonate cercararial predators and the down-stream hosts, diversity effects were observed, albeit depending on odonate density (Rohr et al. 2015). In this case, the effect was not only the result of parasite removal but also of predation on focal hosts by some of the odonate species and non-consumptive predator effect in the form of fear-induced behavioural changes in hosts (Rohr et al. 2015). Such non-consumptive effects of non-host cercarial predators on parasite transmission have also been observed in other trematode species (Orlofske et al. 2014; Koprivnikar and Urichuk 2017). This suggests that the addition of hosts to experimental units adds yet another layer of diversity-mediated effects, further suggesting that diversity-disease relationships are probably highly conditional on the disease system at hand.

Similar non-consumptive effects of non-hosts may also be relevant in our system. In our experiments, we focussed on diversity effects on parasite removal by omitting the down-stream mussel hosts. While this allowed us to disentangle diversity from density effects of non-hosts on parasite removal and to determine the consequences for the exposure risk of down-stream hosts, it does not allow us to make inferences on whether differential parasite removal will lead to differential transmission and infection levels in mussels in the presence of the non-hosts. In general, transmission can always be decomposed into an exposure and a susceptibility component and both have important consequences for parasite transmission (Civitello and Rohr 2014). Hence, if non-hosts do not only affect host exposure but also host susceptibility, this will have consequences for the ultimate transmission success of infective stages. Indeed, such non-consumptive effects on host susceptibility may also play a role in our system. The presence of crabs, perceived by mussels through olfactorial cues, affects mussel behaviour by inducing valve closure to evade predation. This behavioural response to predation cues, in turn, reduces the susceptibility of mussels through reduced filtration which is the major infection route for cercariae, ultimately leading to lower metacercarial infection levels in presence of crab cues (Cornelius et al. 2023). In our experiments, we revealed diversity effects of non-hosts on the pool of infective stages, which is a strong determinant of infection risk through the parasite exposure component of the transmission process. However, taking the focal host and non-consumptive effects of non-hosts on the susceptibility component of the transmission process into account will most likely make diversity effects even more complex. Further complexity will arise from the fact that transmission processes in the field will be embedded in much more complex diversity scenarios than the two-species combinations that we captured with our experimental response surface designs.

With such a complexity of factors modifying the relationship between non-host diversity and parasite exposure and host susceptibility, the question arises to what extent diversity effects observed in relatively simple experimental settings can also be observed in the field. Regarding the parasite-host system investigated in our experiments, large-scale investigations of the correlates of infection levels in mussels living on mixed mussel and oysters beds in our study area did not reveal evidence for dilution effects of oysters on infections of mussels with the trematode species we used in our experiments (Goedknegt et al. 2019). However, this does not mean that non-host diversity-mediated effects do not occur under natural conditions as field experiments in the same area have shown a decrease in infection levels in mussels in the presence of oysters (Thieltges et al. 2009). Instead, it is more likely that the complexity of species interactions with direct and indirect effects on parasite removal and transmission hampers the detection of specific diversity effects in the field. This may be further exacerbated in marine ecosystems, where wide-spread parasite dispersal can occur but may also be subjected to additional mediating effects, such as those caused by tides, ocean currents and other physical dynamics. Studies in much more closed ecosystems such as freshwater lakes and wetlands have been more successful in finding some field-based evidence for non-host diversity-mediated effects on parasite infection levels (Lagrue and Poulin 2015; Rohr et al. 2015). However, whether there really are differences in the relevance and strength of diversity-disease relationships among the major realms still remains to be investigated. Therefore, more studies from different host and disease systems, ideally combining experimental and correlative field approaches as well disentangling the consumptive and non-consumptive effects of non-hosts, are needed to identify any potential general patterns in the direction and strength of diversity effects on parasite transmission.

## Conclusions

Our experiments revealed strong non-host diversity effects on parasite removal. However, these diversity effects did not generally result in a reduction of the number of infective stages, instead the direction and magnitude of changes in parasite removal were non-linear, probably driven by intra- and interspecific interactions among the different non-host species in the experimental communities. Given the likelihood of a wide range of species interactions in natural communities, non-host diversity effects on parasite removal are probably often non-linear and context-dependent. Response surface experimental designs, albeit limited to two non-host scenarios, are a promising approach to unravel the complexity of non-host diversity effects and the underlying mechanisms, ultimately aiming to understand the relationship between community diversity and disease risk.

### Supplementary Information

Below is the link to the electronic supplementary material.

Supplementary file1 (PDF 468 KB)

## Data Availability

The data supporting the results of this research will be archived in a public repository (4tu.nl) and the corresponding DOI will be included in the manuscript upon publication.

## References

[CR1] Bommarito C, Khosravi M, Thieltges DW, Pansch C, Hamm T, Pranovi F, Vajedsamiei J (2022). Combined effects of salinity and trematode infections on the filtration capacity, growth and condition of mussels. Mar Ecol Prog Ser.

[CR2] Burge CA, Closek CJ, Friedman CS, Groner ML, Jenkins CM, Shore-Maggio A, Welsh JE (2016). The use of filter-feeders to manage disease in a changing world. Integr Comp Biol.

[CR3] Cardinale BJ, Duffy J, Gonzalez A, Hooper DU, Perrings C, Venail P, Narwani A, Mace GM, Tilman D, Wardle AD, Kinzig AP, Daily GC, Loreau M, Grace JB, Larigauderie A, Srivastava DS, Naeem S (2012). Biodiversity loss and its impact on humanity. Nature.

[CR4] Cardinale BJ, Srivastava DS, Duffy JE, Wright JP, Downing AL, Sankaran M, Jouseau C (2006). Effects of biodiversity on the functioning of trophic groups and ecosystems. Nature.

[CR5] Civitello DJ, Rohr JR (2014). Disentangling the effects of exposure and susceptibility on transmission of the zoonotic parasite *Schistosoma mansoni*. J Anim Ecol.

[CR6] Civitello DJ, Cohen J, Fatima H, Halstead NT, Liriano J, McMahon TA, Ortega CN, Sauer EL, Sehgal T, Young S, Rohr JR (2015). Biodiversity inhibits parasites: broad evidence for the dilution effect. Proc Natl Acad Sci.

[CR7] Cohen JM, Civitello DJ, Brace AJ, Feichtinger EM, Ortega CN, Richardson JC, Sauer EL, Liu X, Rohr JR (2016). Spatial scale modulates the strength of ecological processes driving disease distributions. Proc Natl Acad Sci.

[CR8] Cornelius A, Buschbaum C, Khosravi M, Waser AM, Wegner KM, Thieltges DW (2023). Effect of predation risk on parasite transmission from first to second intermediate trematode hosts. J Anim Ecol.

[CR9] Correia S, Freitas R, de Montaudouin X, MagalhãesL, (2021). Effect of light on the trematode *Himasthla elongata*: from cercarial behaviour to infection success. Dis Aquat Org.

[CR10] Duffy JE, Godwin CM, Cardinale BJ (2017). Biodiversity effects in the wild are common and as strong as key drivers of productivity. Nature.

[CR11] Goedknegt MA, Nauta R, Markovic M, Buschbaum C, Folmer EO, Luttikhuizen PC, van der Meer J, Waser AM, Wegner KM, Thieltges DW (2019). How invasive oysters can affect parasite infection patterns in native mussels on a large spatial scale. Oecologia.

[CR12] Goedknegt MA, Welsh JE, Drent J, Thieltges DW (2015). Climate change and parasite transmission: how temperature affects parasite infectivity via predation on infective stages. Ecosphere.

[CR13] Griffen BD, Byers JE (2006). Partitioning mechanisms of predator interference in different habitats. Oecologia.

[CR14] Halliday FW, Rohr JR (2019). Measuring the shape of the biodiversity-disease relationship across systems reveals new findings and key gaps. Nat Comm.

[CR64] Halliday FW, Rohr JR, Laine A-L (2020). Biodiversity loss underlies the dilution effect of biodiversity. Ecol Lett.

[CR15] Halsey S (2019). Defuse the dilution effect debate. Nat Ecol Evol.

[CR16] Hooper DU, Chapin FS, Ewel JJ, Inchausti P, Lavorel S, Lawton JH, Lodge DM, Loreau M, Naeem S, Schimid B, Setälä H, Symstad AJ, Vandermeer J, Wardle DA (2005). Effects of biodiversity on ecosystem functioning: a consensus of current knowledge. Ecol Monogr.

[CR17] Hopper JV, Poulin R, Thieltges DW (2008). Buffering role of the intertidal anemone *Anthopleura aureoradiata* in cercarial transmission from snails to crabs. J Exp Mar Biol Ecol.

[CR18] Huang ZYX, Yu Y, Van Langevelde F, De Boer WF (2017). Does the dilution effect generally occur in animal diseases?. Parasitology.

[CR19] Inouye BD (2001). Response surface experimental designs for investigating interspecific competition. Ecology.

[CR20] Johnson PTJ, Dobson A, Lafferty KD, Marcogliese DJ, Memmott J, Orlofske SA, Poulin R, Thieltges DW (2010). When parasites become prey: ecological and epidemiological significance of eating parasites. Trends Ecol Evol.

[CR21] Johnson PTJ, Hartson R, Larson DJ (2008). Diversity and disease : Community structure drives parasite transmission and host fitness. Ecol Lett.

[CR22] Johnson PTJ, Hartson RB, Larson DJ, Sutherland DR (2008). Diversity and disease: community structure drives parasite transmission and host fitness. Ecol Lett.

[CR23] Johnson PTJ, Ostfel RS, Keesing F (2015). Frontiers in research on biodiversity and disease. Ecol Lett.

[CR24] Johnson PTJ, Preston DL, Hoverman JT, Richgels KLD (2013). Biodiversity decreases disease through predictable changes in host community competence. Nature.

[CR25] Johnson PTJ, Thieltges DW (2010). Diversity, decoys and the dilution effect: how ecological communities affect disease risk. J Exp Biol.

[CR26] Keesing F, Holt RD, Ostfeld RS (2006). Effects of species diversity on disease risk. Ecol Lett.

[CR27] Keesing F, Ostfeld RS (2021). Dilution effects in disease ecology. Ecol Lett.

[CR28] Klohmann CA, Padilla-Gamiño JL (2022). Pathogen filtration: an untapped ecosystem service. Front Mar Sci.

[CR29] Koprivnikar J, Urichuk TMY (2017). Time-lagged effect of predators on tadpole behaviour and parasite infection. Biol Let.

[CR30] Koprivnikar J, Thieltges DW, Johnson PTJ (2023). Consumption of trematode parasite infectious stages: from conceptual synthesis to future research agenda. J Helminthol.

[CR31] Lafferty KD, Wood CL (2013). It’s a myth that protection against disease is a strong and general service of biodiversity conservation: response to Ostfeld and Keesing. Trends Ecol Evol.

[CR32] Lagrue C, Poulin R (2015). Local diversity reduces infection risk across multiple freshwater host-parasite associations. Freshw Biol.

[CR33] Lamb JB, van de Water JAJM, Bourne DG, Altier C, Hein MY, Fiorenza EA, Abu N, Jompa J, Harvell CD (2017). Seagrass ecosystems reduce exposure to bacterial pathogens of humans, fishes, and invertebrates. Science.

[CR34] Liddell C, Welsh JE, van der Meer J, Thieltges DW (2017). Effect of dose and frequency of exposure to infectious stages on trematode infection intensity and success in mussels. Dis Aquat Org.

[CR35] Mascaró M, Seed R (2000). Foraging behavior of *Carcinus maenas* (L.): comparisons of size-selective predation on four species of bivalve prey. J Shellfish Res.

[CR36] Mascaro M, Seed R (2001). Choice of prey size and species in *Carcinus maenas* (L.) feeding on four bivalves of contrasting shell morphology. Hydrobiologia.

[CR37] Nikolaev KE, Vladimir V, Prokofiev VV, Levakin IA, Galaktionov KV (2017). How the position of mussels at the intertidal lagoon affects their infection with the larvae of parasitic flatworms (Trematoda: Digenea): a combined laboratory and field experimental study. J Sea Res.

[CR38] Orlofske SA, Jadin RC, Preston DL, Johnson PT (2012). Parasite transmission in complex communities: predators and alternative hosts alter pathogenic infections in amphibians. Ecology.

[CR39] Orlofske SA, Jadin RC, Hoverman JT, Johnson PT (2014). Predation and disease: understanding the effects of predators at several trophic levels on pathogen transmission. Freshw Biol.

[CR40] Orlofske SA, Jadin RC, Johnson PTJ (2015). It's a predator-eat-parasite world: how characteristics of predator, parasite and environment affect consumption. Oecologia.

[CR41] Ostfeld RS, Keesing F (2000). Biodiversity and disease risk: the case of Lyme disease. Conserv Biol.

[CR42] Ostfeld RS, Keesing F (2012). Effects of host diversity on infectious disease. Annu Rev Ecol Evol Syst.

[CR43] Ostfeld RS, Keesing F (2013). Straw men don’t get Lyme disease: response to Wood and Lafferty. Trends Ecol Evol.

[CR44] R Core Team (2019) R: a language and environment for statistical computing. R Foundation for Statistical Computing, Vienna, Austria

[CR46] Randolph SE, Dobson A (2012). Pangloss revisited: a critique of the dilution effect and the biodiversity-buffers-disease paradigm. Parasitology.

[CR47] Rohr JR, Civitello DJ, Crumrine PW, Halstea NT, Miller AD, Schotthoefer AM, Stenoien C, Johnson LB, Beasley VR (2015). Predator diversity, intraguild predation, and indirect effects drive parasite transmission. Proc Natl Acad Sci.

[CR48] Rohr JR, Civitello DJ, Halliday FW, Hudson PJ, Lafferty KD, Wood CL, Mordecai EA (2020). Towards common ground in the biodiversity-disease debate. Nat Ecol Evol.

[CR45] RStudio Team (2018) RStudio: integrated Development for R. RStudio, PBC, Boston, USA

[CR49] Salkeld DJ, Padgett KA, Jones JH (2013). A meta-analysis suggesting that the relationship between biodiversity and risk of zoonotic pathogen transmission is idiosyncratic. Ecol Lett.

[CR50] Searle CL, Biga LM, Spatafora JW, Blaustein AR (2011). A dilution effect in the emerging amphibian pathogen *Batrachochytrium dendrobatidis*. Proc Natl Acad Sci.

[CR51] Smallegange IM, van der Meer J, Kurvers RHJM (2006). Disentangling interference competition from exploitative competition in a crab-bivalve system using a novel experimental approach. Oikos.

[CR52] Sneddon LU, Taylor AC, Huntingford FA, Watson DG (2000). Agonistic behaviour and biogenic amines in shore crabs *Carcinus maenas*. J Exp Biol.

[CR53] Stier T, Drent J, Thieltges DW (2015). Trematode infections reduce clearance rates and condition in blue mussels *Mytilus edulis*. Mar Ecol Prog Ser.

[CR54] Thieltges DW (2006). Effect of infection by the metacercarial trematodes *Renicola roscovita* on growth in intertidal blue mussel *Mytilus edulis*. Mar Ecol Prog Ser.

[CR55] Thieltges DW, Jensen KT, Poulin R (2008). The role of biotic factors in the transmission of free-living endohelminth stages. Parasitology.

[CR56] Thieltges DW, Reise K, Prinz K, Jensen KT (2009). Invaders interfere with native parasite–host interactions. Biol Invasions.

[CR57] Tilman D, Isbell F, Cowles JM (2014). Biodiversity and ecosystem functioning. Annu Rev Ecol Evol Syst.

[CR60] Welsh JE, van der Meer J, Brussaard CPD, Thieltges DW (2014). Inventory of organisms interfering with transmission of a marine trematode. J Mar Biol Assoc U K.

[CR58] Welsh JE, Hempel A, Markovic M, van der Meer J, Thieltges DW (2019). Consumer and host body size effects on the removal of trematode cercariae by ambient communities. Parasitology.

[CR59] Welsh JE, Steenhuis P, Ribeiro de Morae K, van der Meer J, Thieltges DW, Brussaard CPD (2020). Marine virus predation by non-host organisms. Sci Rep.

[CR61] Welsh JE, Markovic M, van der Meer J, Thieltges DW (2023) Data presented in the paper ‘Complex effects of non-host diversity on the removal of free-living infective stages of parasites’. 4TU ResearchData.Dataset, Netherlands. 10.4121/64220d63-9bbf-454a-a82e-b414eb11da69

[CR62] Werding B (1969). Morphologie, Entwicklung und Ökologie digener Trematodenlarven der Strandschnecke *Littorina littorea*. Mar Biol.

[CR63] Wood CL, Lafferty KD (2013). Biodiversity and disease: a synthesis of ecological perspectives on Lyme disease transmission. Trends Ecol Evol.

