# Supplementary material for: Non-linear effects of non-host diversity on the removal of free-living infective stages of parasites
Source: Oecologia. 2024 Feb 1;204(2):339–49. doi: 10.1007/s00442-023-05462-2 (PMC10907414; doi:10.1007/s00442-023-05462-2)
Supplement: Supplementary file 1 — Supplementary file1 (PDF 468 KB) [file 442_2023_5462_MOESM1_ESM.pdf]

Electronic Supplemental Material (ESM)

Non-linear effects of non-host diversity on the removal of free-living infective stages of parasites

Jennifer E. Welsh[1)], Mirjana Markovic[1)], Jaap van der Meer[2,3)], David. W. Thieltges[1,4)]

1) Department of Coastal Systems, NIOZ Royal Netherlands Institute for Sea Research, PO Box 59, 1790 AB Den Burg, The Netherlands

2) Wageningen Marine Research, Korringaweg 7, 4401 NT, Yerseke, The Netherlands

3) Aquaculture and Fisheries Group, Wageningen University and Research, Wageningen

4) Groningen Institute for Evolutionary Life-Sciences, GELIFES, University of Groningen, Nijenborgh 7, 9747 AG Groningen, the Netherlands

Corresponding author: Davd.Thieltges@nioz.nl

ESM 1 Background information on field situation

In the European Wadden Sea, the study area where our experiments were conducted, Pacific oysters (*Crassostrea gigas*) form extensive beds on the top of the sedimentary tidal flats (Figure S1a). The densely packed oysters construct a reef-like structure that is inhabited by blue mussels (*Mytilus edulis*) and periwinkles (*Littorina littorea*), the first and second intermediate host, respectively, of the trematode parasite species studied (Figure S1d,e). The algae *Sargassum muticum* can locally cover entire mussel/oyster beds at high densities (Figure S1b,c) and shore crabs (*Carcinus maenas*) live within the habitat matrix created by oysters and mussels (Figure S1f). On many oyster/mussel beds tide pools occur that also hold water at low tide, creating situations of high densities of organisms at relatively small water volumes (Figure S1a-c). For more information on oyster/mussel beds please see Folmer et al. (2017).

Folmer E., Büttger H., Herlyn M., Markert A., Millat G., Troost K. & Wehrmann A. (2017) Beds of blue mussels and Pacific oysters. In: Wadden Sea Quality Status Report. Eds.: Kloepper S. et al., Common Wadden Sea Secretariat, Wilhelmshaven, Germany. Last updated 01.03.2018. qsr.waddensea-worldheritage.org/reports/beds-of-blue-mussels-and-pacific-oysters

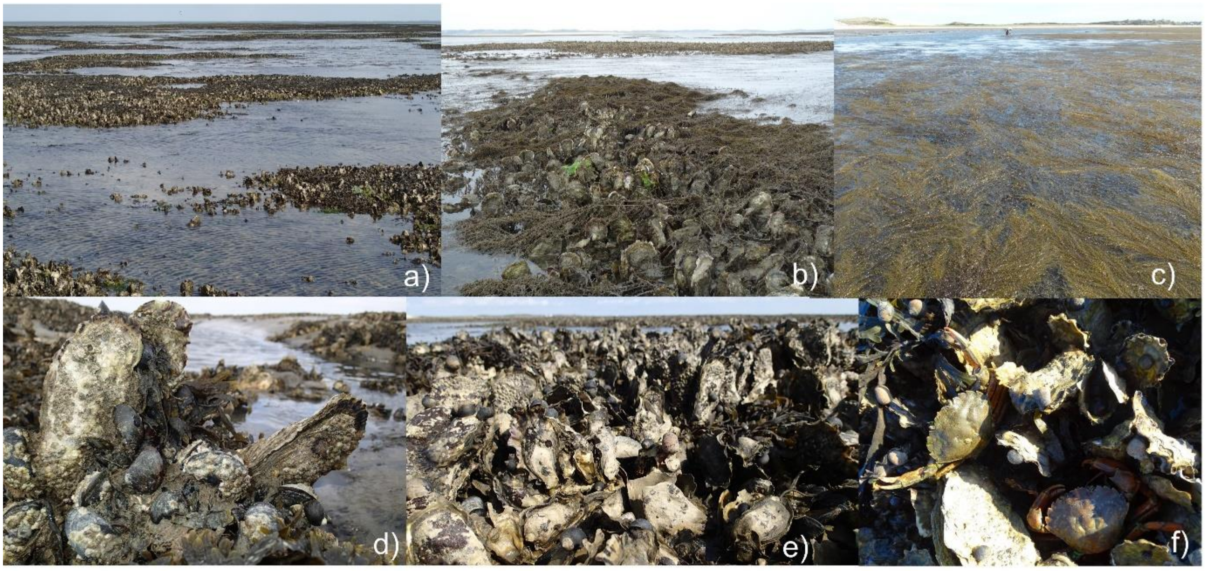

Fig. S1 Mixed beds of Pacific oysters (*Crassostrea gigas*) and blue mussels (*Mytilus edulis*) in the study area (a), the European Wadden Sea, which can locally be entirely covered by the algae *Sargassum muticum* (b,c). Within the oyster/mussel matrix and in tide pools (a-c), blue mussels (*Mytilus edulis*) and periwinkles (*Littorina littorea*), which serve as intermediate hosts for the parasite species studied, as well as shore crabs (*Carcinus maenas*) occur at high densities (d-e). Photo copyrights: a,d,e) David Thieltges; b,c) Christian Buschbaum; f) Karsten Reise

Table S1 Output tables from the best-fitting models, which were the most complex ones (i.e. including the interaction between both non-host species: $\theta_{i,j}=\mu+\alpha_i+\beta_j+\gamma_{i,j}$, where i=0,...,3 and j=0,...3) in all three experiments. The matrix design for the output tables is given in the top table. Note that, for example, $\theta_{0,0}=\mu$ and $\theta_{0,2}=\mu+\beta_2$ and $\theta_{3,2}=\mu+\alpha_3+\beta_2+\gamma_{3,2}$. For further details of the statistical approach see main text

**Matrix design**	Density level species A			
Density level species B	0	1	2	3
0	μ	α_1	α_2	α_3
1	$ß_1$	$\gamma_{1,1}$	$\gamma_{2,1}$	$\gamma_{3,1}$
2	$ß_2$	$\gamma_{1,2}$	$\gamma_{2,2}$	$\gamma_{3,2}$
3	$ß_3$	$\gamma_{1,3}$	$\gamma_{2,3}$	$\gamma_{3,3}$

**Crab-seaweed**	Crab density			
Seaweed density	0	1	2	3
0	-0.0151	-0.0902	-0.2593	-0.2996
5	-0.2143	0.0419	-0.1556	-0.1346
15	-0.2996	0.0902	-0.0515	-0.3867
30	-0.0388	0.0163	-0.133	-0.9106

**Seaweed-oyster**	Seaweed density			
Oyster density	0	5	15	30
0	-0.0305	-0.0945	-0.135	-0.4316
1	-0.4316	0.3581	0.355	0.4276
2	-0.6727	0.4482	0.5929	0.6727
6	-0.9048	0.5557	0.6992	0.8766

**Oyster-crab**	Oyster density			
Crab density	0	1	2	6
0	-0.0279	-0.4106	-0.8637	-1.886
1	-0.3041	0.5132	0.4137	0.9887
2	-0.4788	0.3854	0.7861	1.5545
3	-0.4342	0.6983	0.8755	1.6649

Table S2 Removal rate and cercarial survival (%) for each diversity and density combination of seaweed and crabs as extracted from the best-fitting model (model 1; see Table 1). X_1 and X_2 refer to the factor coding used in the model selection procedure (see Fig. S1). N = 4 per treatment combination

**Non-host density**			
**Seaweed (X_1)**	**Crabs (X_2)**	**Removal rate**	**Cercarial survival (%)**
0	0	0.02	98.5%
0	1	0.23	79.5%
0	2	0.31	73.0%
0	3	0.05	94.8%
5	0	0.11	90.0%
5	1	0.28	75.8%
5	2	0.31	73.0%
5	3	0.13	88.0%
15	0	0.27	76.0%
15	1	0.64	52.5%
15	2	0.63	53.5%
15	3	0.45	64.0%
30	0	0.31	73.0%
30	1	0.66	51.5%
30	2	1.00	36.8%
30	3	1.26	28.3%

Table S3 Removal rate and cercarial survival (%) for each diversity and density combination of oysters and seaweed as extracted from the best-fitting model (model 1; see Table 1). X_1 and X_2 refer to the factor coding used in the model selection procedure (see Fig. S1). N = 4 per treatment combination

**Non-host density**			
**Oysters (X_1)**	**Seaweed (X_2)**	**Removal rate**	**Cercarial survival (%)**
0	0	0.03	97.0%
1	0	0.46	63.0%
2	0	0.70	49.5%
6	0	0.94	39.3%
0	5	0.12	88.3%
1	5	0.20	82.0%
2	5	0.35	70.5%
6	5	0.47	62.3%
0	15	0.17	84.8%
1	15	0.24	78.5%
2	15	0.25	78.3%
6	15	0.37	69.0%
0	30	0.46	63.0%
1	30	0.47	62.8%
2	30	0.46	63.0%
6	30	0.49	61.3%

Table S4 Removal rates and cercarial survival (%) for each diversity and density combination of oysters and crabs as extracted from the best-fitting model (model 1; see Table 1). X_1 and X_2 refer to the factor coding used in the model selection procedure (see Fig. S1). N = 4 per treatment combination

**Non-host density**			
**Oysters (X_1)**	**Crabs (X_2)**	**Removal rate**	**Cercarial survival (%)**
0	0	0.03	97.3%
0	1	0.33	71.8%
0	2	0.51	60.3%
0	3	0.46	63.0%
1	0	0.44	64.5%
1	1	0.23	79.5%
1	2	0.53	58.8%
1	3	0.17	84.0%
2	0	0.89	41.0%
2	1	0.78	45.8%
2	2	0.58	55.8%
2	3	0.45	63.8%
6	0	1.91	14.8%
6	1	1.23	29.3%
6	2	0.84	43.2%
6	3	0.68	50.5%

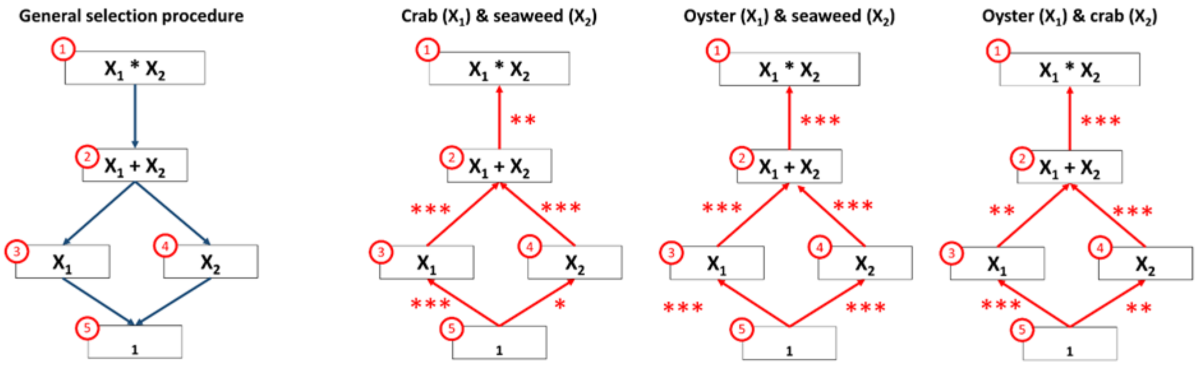

Fig. S2 General model selection procedure followed for testing for significant differences between the different models relating the two non-host species (X_1 and X_2), starting with the most complex model (1) down to the simplest model (5), and the respective pathways and significance results for each of the three non-host combinations: crabs and seaweed, seaweed and oysters, and oysters and crabs. Asterisks denote significance levels: '***' 0.001; '**' 0.01; '*' 0.05; '.' 0.1; ' ' 1. For details see main text
